# Augmented renal clearance in Japanese intensive care unit patients: a prospective study

**DOI:** 10.1186/s40560-016-0187-7

**Published:** 2016-10-03

**Authors:** Yasumasa Kawano, Shinichi Morimoto, Yoshito Izutani, Kentaro Muranishi, Hironari Kaneyama, Kota Hoshino, Takeshi Nishida, Hiroyasu Ishikura

**Affiliations:** Department of Emergency and Critical Care Medicine, Fukuoka University Hospital, Faculty of Medicine, 7-45-1 Nanakuma, Jonan-ku, Fukuoka, 8140180 Japan

**Keywords:** Augmented renal clearance, Intensive care unit, Japan, Risk factor, eGFR

## Abstract

**Background:**

Augmented renal clearance (ARC) of circulating solutes and drugs has been recently often reported in intensive care unit (ICU) patients. However, only few studies on ARC have been reported in Japan. The aims of this pilot study were to determine the prevalence and risk factors for ARC in Japanese ICU patients with normal serum creatinine levels and to evaluate the association between ARC and estimated glomerular filtration rate (eGFR) calculated using the Japanese equation.

**Methods:**

We conducted a prospective observational study from May 2015 to April 2016 at the emergency ICU of a tertiary university hospital; 111 patients were enrolled (mean age, 67 years; interquartile range, 53–77 years). We measured 8-h creatinine clearance (CL_CR_) within 24 h after admission, and ARC was defined as body surface area-adjusted CL_CR_ ≥ 130 mL/min/1.73 m^2^. Multiple logistic regression analysis was performed to identify the risk factors for ARC. Moreover, a receiver operating curve (ROC) analysis, including area under the receiver operating curve (AUROC) was performed to examine eGFR accuracy and other significant variables in predicting ARC.

**Results:**

In total, 43 patients (38.7 %) manifested ARC. Multiple logistic regression analysis was performed for age, body weight, body height, history of diabetes mellitus, Acute Physiology and Chronic Health Evaluation II scores, admission categories of post-operative patients without sepsis and trauma, and serum albumin, and only age was identified as an independent risk factor for ARC (odds ratio, 0.95; 95 % confidence interval [CI], 0.91–0.98). Moreover, the AUROC of ARC for age and eGFR was 0.81 (95 % CI, 0.72–0.89) and 0.81 (95 % CI, 0.73–0.89), respectively. The optimal cutoff values for detecting ARC were age and eGFR of ≤63 years (sensitivity, 72.1 %; specificity, 82.4 %) and ≥76 mL/min/1.73 m^2^ (sensitivity, 81.4 %; specificity, 72.1 %), respectively.

**Conclusions:**

ARC is common in Japanese ICU patients, and age was an independent risk factor for ARC. In addition, age and eGFR calculated using the Japanese equation were suggested to be useful screening tools for identifying Japanese patients with ARC.

## Background

Clinicians often modify drug prescriptions to a patient’s glomerular filtration rate (GFR) because renal clearance influences the pharmacokinetics of many commonly prescribed agents [1]. Intensive care unit (ICU) patients in a critical condition with severe morbidity sometimes experience acute kidney injury (AKI) [2]. Clinicians usually reduce drug doses to prevent drug toxicity because drug elimination is impaired in these patients [3]. In contrast, recent studies [1, 4] reported that the phenomenon of increased renal blood flow due to an increased cardiac output might lead to an augmented renal clearance (ARC) of circulating solutes and drugs. Although creatinine clearance (CL_CR_) is not a gold standard measurement of GFR (such as inulin clearance), a close correlation was found between the ARC phenomenon and CL_CR_ [5, 6], and ARC phenomenon is characterized by CL_CR_ ≥ 130 mL/min/1.73 m^2^ [7]. ARC is potentially related to insufficient treatment and poor prognosis due to sub-therapeutic drug concentrations particularly in critically ill patients [5, 6, 8, 9]; therefore, ARC should be recognized in the ICU setting. However, ARC may occur in patients with normal serum creatinine (*S*_Cr_) level [10, 11], and CL_CR_ measurement is not routinely performed in the ICU for daily treatments; the accurate recognition of this phenomenon is difficult for clinicians. For this reason, previous studies [12, 13] verified the correlation between ARC and estimated glomerular filtration rate (eGFR), which was calculated using various formulas (such as Cockcroft–Gault equation [14], Modification of Diet in Renal Disease [MDRD] Study equation [15], Robert equation [16], and the Chronic Kidney Disease Epidemiology Collaboration [CKD-EPI] equation [17]) used in clinical practice worldwide. In contrast, few studies and discussions regarding ARC in Japan have been reported. To the best of our knowledge, no study has been reported on the correlation between ARC and eGFR calculated using the Japanese eGFR equation, which is used throughout Japan [18]. The aims of this pilot study were to determine the prevalence and risk factors for ARC in Japanese ICU patients with normal S_Cr_ levels and to evaluate the association between ARC and eGFR calculated using the Japanese equation.

## Methods

### Setting

This prospective, single-center, observational study was conducted in a 32-bed emergency ICU of the Fukuoka University Hospital, a tertiary hospital in Japan, from May 2015 to April 2016. This study was approved by the institutional ethics committee (number 15-4-07), and informed consent was obtained from all participants or a surrogate decision maker.

### Study population

Patients who were expected to stay more than 24 h, with no evidence of renal impairment (admission *S*_Cr_ > 1.1 mg/dL) and no history of renal replacement therapy were enrolled. The exclusion criteria for study admission were as follows: age < 18 years, pregnancy, suspicion of rhabdomyolysis or admission S_Cr_ kinase concentration >5000 IU/L, diagnosis of cardiopulmonary arrest on admission, and developing AKI as defined by the Risk, Injury, Failure, Loss of kidney function, End-Stage Kidney Disease criteria [19]. Moreover, patients treated without both an intra-arterial cannula, and an indwelling urinary catheter (IDC) were also excluded. In total, 111 patients were enrolled.

### Data collection and definition

Demographic and laboratory data, including age, sex, body measurements, cumulative number of systemic inflammatory response syndrome (SIRS) [20], medical history of diabetes mellitus, and the levels of serum albumin and blood glucose were recorded on admission. Information regarding ventilation variables, vasopressor or inotrope administration, diuretic use, and admission diagnosis was recorded after the first 24 h. In addition, the patients were divided into the following four groups based on the diagnosis on admission: sepsis, post-operative patients without sepsis, trauma (divided based on severity, injury severity score [ISS] ≥ 16 or ISS < 16), and others.

Physiological and laboratory data needed to calculate the Acute Physiology and Chronic Health Evaluation (APACHE) II scores and Sequential Organ Failure Assessment (SOFA) scores were reported as the worst value within 24 h after hospital admission. The mean urine output (mL/kg/h) and fluid balance were recorded during the first hospital day. Because previous reports [21, 22] suggest that renal function can be measured most accurately using an 8-, 12-, or 24-h CL_CR_ collection, the 8-h CL_CR_ was measured in this study. Urinary volume was measured from the IDC within the first 24 h of admission, and the blood sampling for eGFR and CL_CR_ measurement were performed simultaneously after the completion of the 8-h CL_CR_ collection. The urinary creatinine (*U*_Cr_) level and the *S*_Cr_ were determined by laboratory analysis by using an enzymatic method.

We calculated eGFR by using a three-variable Japanese equation [18].

For males: eGFR (mL/min/1.73 m^2^) = 194 × [*S*_Cr_(mg/dL)]^‐ 1.094^ × age^‐ 0.287^

For females: eGFR (mL/min/1.73 m^2^) = 194 × [*S*_Cr_(mg/dL)]^‐ 1.094^ × age^‐ 0.287^ × 0.739

The CL_CR_ was calculated by using the standard formula. CL_CR_ values were subsequently normalized to a body surface area (BSA) of 1.73 m^2^ as per convention.

CL_CR_ and BSA were calculated based on the following formulae:

$$ \begin{array}{l}\mathrm{C}{\mathrm{L}}_{\mathrm{CR}}\left(\mathrm{mL}/ \min /1.73\ {\mathrm{m}}^2\right) = \left[{U}_{\mathrm{Cr}}\left(\mathrm{mg}/\mathrm{dL}\right)/{S}_{\mathrm{Cr}}\left(\mathrm{mg}/\mathrm{dL}\right)\left] \times \right[8\hbox{-} \mathrm{h}\ \mathrm{urinary}\;\mathrm{volume}\left(\mathrm{mL}\right)/480\left] \times \right[1.73/\mathrm{B}\mathrm{S}\mathrm{A}\left({\mathrm{m}}^2\right)\right]\\ {}\mathrm{B}\mathrm{S}\mathrm{A}\left({\mathrm{m}}^2\right) = 0.007184 \times {\left[\mathrm{height}\ \left(\mathrm{cm}\right)\right]}^{0.725} \times {\left[\mathrm{weight}\ \left(\mathrm{kg}\right)\right]}^{0.425}\end{array} $$

Data collection began immediately after obtaining an informed consent and was discontinued at ICU discharge or death, development of severe renal impairment (measured CL_CR_ < 30 mL/min/1.73 m^2^), initiation of renal replacement therapy, intra-arterial cannula or IDC removal, and patient consent withdrawal. ARC was defined as an 8-h CL_CR_ ≥ 130 mL/min/1.73 m^2^ [7].

### Statistical analysis

Continuous data were expressed as mean (standard deviation [SD]) or median (interquartile range [IQR]), and categorical data as percentage. The Student *t* test or Mann–Whitney *U* test and chi-square test were used for continuous and categorical data, respectively. Multiple logistic regression analysis was performed to identify the risk factors for ARC. Because serum albumin levels and diabetic conditions were shown to influence tubular creatinine secretion [23, 24], these factors were included as explanatory variables in multivariate analysis. Furthermore, the explanatory variables in this analysis were also determined from any variables with a *p* value of less than 0.05 in the univariate analysis. The odds ratio (OR) and 95 % confidence interval (CI) were calculated. The correlations between the measured CL_CR_ and eGFR were assessed by using Spearman correlation coefficient (*r*), and the Bland and Altman method [25] was used to check the bias and limits of agreement between the measured CL_CR_ and eGFR. Bias was defined as the mean difference between eGFR and measured CL_CR_. The 95 % limits of agreement were calculated as the bias ±1.96 SD. Moreover, a receiver operating curve (ROC) analysis, including the area under the receiver operating curve (AUROC), was performed to examine the accuracy of the eGFR and other significant variables in predicting ARC. The ROC was plotted for each score by using sensitivity and specificity values for true prediction of ARC across the entire range of potential cutoff values to predict ARC. The AUROC was constructed and compared as described in a previous report [26]. All tests were two-tailed, and a *p* value of <0.05 was considered statistically significant.

All statistical analyses were performed by using the EZR software program (Saitama Medical Center, Jichi Medical University, Saitama, Japan) [27], which is a graphical user interface for the R software program (The R Foundation for Statistical Computing, Vienna, Austria). More precisely, it is a modified version of R commander, which was designed to add statistical functions frequently used in biostatistics.

## Results

### Baselines characteristics of study subjects

The characteristics of the enrolled patients are shown in Table 1.

**Table 1:** Demographic and laboratory data

Variable	All patients (*n* = 111)	Patients with ARC (*n* = 43)	Patients without ARC (*n* = 68)	*p* value^a^
Age, median (IQR)	67 (53–77)	55 (38–65)	72 (66–79)	<0.05
Male sex, *n* (%)	62 (55.9)	22 (51.2)	40 (58.8)	0.44
Body weight (kg), median (IQR)	56.3 (49.9–68.2)	60.7 (52.8–74.1)	53.2 (47.9–62.5)	<0.05
Body height (m), mean (SD)	1.61 (0.1)	1.64 (0.1)	1.59 (0.09)	<0.05
Body mass index (kg/m^2^), mean (SD)	22.7 (3.88)	23.6 (3.75)	22.1 (3.87)	<0.05
Body surface area (m^2^), median (IQR)	1.57 (1.46–1.79)	1.67 (1.54–1.85)	1.55 (1.41–1.69)	<0.05
Diabetes mellitus, *n* (%)	22 (19.8)	5 (11.6)	17 (25)	0.09
Mechanical ventilation, *n* (%)	21 (18.9)	6 (14)	15 (22.4)	0.33
Vasopressor, *n* (%)	2 (1.8)	0	2 (2.9)	0.52
Inotrope, *n* (%)	8 (7.2)	2 (4.6)	6 (8.8)	0.48
Diuretic therapy, *n* (%)	6 (5.4)	1 (2.3)	5 (7.4)	0.4
APACHE II scores, median (IQR)	14 (10.5–19.5)	13 (8.5–15.5)	16 (11.8–23)	*<*0.05
SOFA scores, median (IQR)	3 (2–5)	3 (2–4)	3 (2–5)	0.33
The cumulative number of SIRS, median (IQR)	1 (1–2)	1 (1–2)	1 (1–2)	0.96
Admission category, *n* (%)				
Sepsis^b^	3 (2.7)	0	3 (4.4)	0.28
Post-operative patients without sepsis	25 (22.5)	4 (9.3)	21 (30.9)	*<*0.05
Trauma	32 (28.8)	20 (46.5)	12 (17.6)	*<*0.05
ISS ≥ 16	19	10	9	
ISS < 16	13	10	3	
Others	51 (45.9)	19 (44.2)	32 (47.1)	0.85
Serum albumin (g/dL), median (IQR)	3.9 (3.4–4.3)	4.2 (3.7–4.4)	3.8 (3.2–4.2)	*<*0.05
Blood glucose (mg/dL), median (IQR)	136 (115–160)	128 (111–150)	141 (118–168)	0.12
Mean urine output (mL/kg/h), median (IQR)	0.92 (0.64–1.36)	0.94 (0.7–1.4)	0.77 (0.6–1.35)	0.29
Fluid balance (mL), median (IQR)	739 (55.5–1290)	993 (−70–1460)	572 (81.3–1125)	0.33

*ARC* augmented renal clearance, *IQR* interquartile range, *SD* standard deviation, *APACHE* Acute Physiology and Chronic Health Evaluation, *SOFA* Sequential Organ Failure Assessment, *SIRS* systemic inflammatory response syndrome, *ISS* injury severity score

^a^The *p* values were evaluated by comparison between patients with and without ARC

^b^Sepsis was diagnosed based on evidence of infection along with the presence of SIRS

We enrolled 111 patients in this study (mean age, 67 years [IQR, 53–77 years], 55.9 % male). Of these, 43 patients (38.7 %) were identified as manifesting ARC. In addition, ARC occurred more frequently in trauma patients (20/32, 62.5 %) and less frequently in post-operative patients without sepsis (4/25, 16.0 %), in comparison with the overall incidence of 38.7 % (43/111). The mean APACHE II score was 14 (IQR, 10.5–19.5), and the mean SOFA score was 3 (IQR, 2–5). Vasopressor and diuretic therapies were administered to a few patients in this study. Moreover, few patients had an admission diagnosis of sepsis (2.7 %), and only 59.4 % (19/32) were categorized as severe trauma patients (ISS ≥ 16).

### Risk factors for ARC

The following variables were significantly different between patients with and without ARC: age, body weight, body height, body mass index, BSA, APACHE II scores, admission categories of post-operative patients without sepsis and trauma, and serum albumin (all *p* < 0.05). Multiple logistic regression analysis was performed for eight variables (such as age, body weight, body height, history of diabetes mellitus, APACHE II scores, admission categories of post-operative patients without sepsis and trauma, and serum albumin), and the result showed that only age is an independent risk factor for ARC (OR, 0.95; 95 % CI, 0.91–0.98) (Table 2).

**Table 2:** Multiple logistic regression analysis for augmented renal clearance

Variables	OR (95 % CI)	*p* value
Age	0.95 (0.91–0.98)	<0.05
Body weight	1.03 (0.98–1.09)	0.25
Body height	0.96 (0.89–1.02)	0.21
Diabetes mellitus	0.73 (0.20–2.73)	0.64
APACHE II scores	0.95 (0.88–1.03)	0.24
Post-operative patients without sepsis	0.28 (0.07–1.04)	0.06
Trauma	1.83 (0.60–5.59)	0.29
Serum albumin	1.36 (0.63–2.93)	0.44

*OR* odds ratio, *CI* confidence interval, *APACHE* Acute Physiology and Chronic Health Evaluation

### Evaluation of eGFR calculated using the Japanese equation

**Fig. 1:**
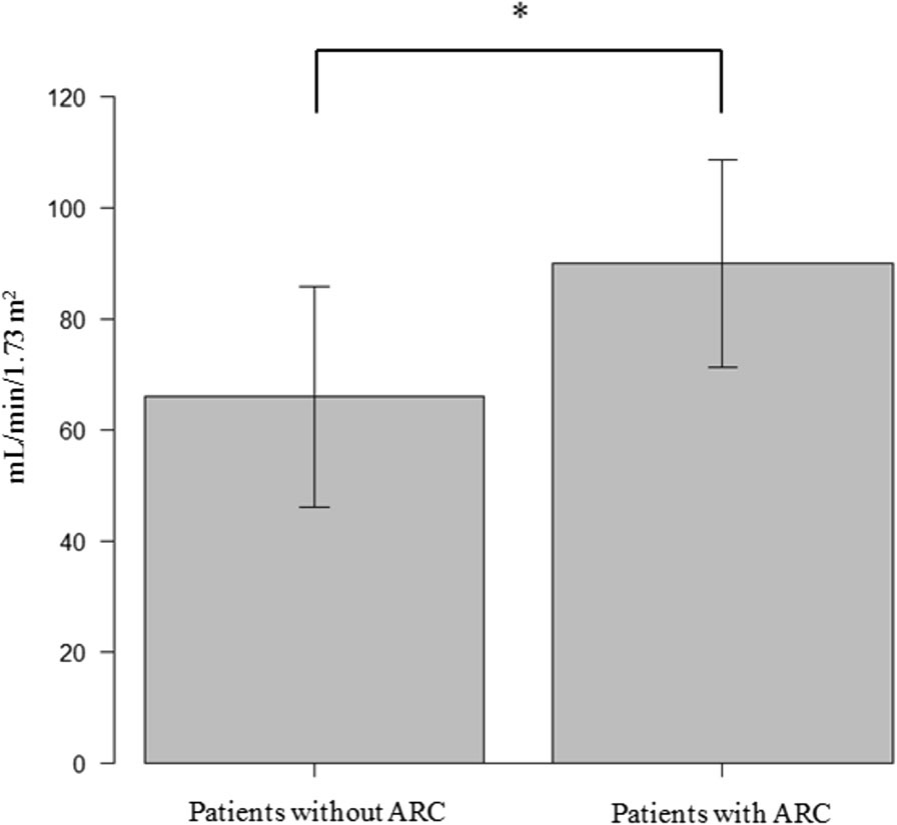
Comparison of the estimated glomerular filtration rate (eGFR) in patients with and without augmented renal clearance (ARC). The eGFR in patients with ARC was significantly higher than that in patients with ARC (*p* < 0.05) * *p* < 0.05

**Fig. 2:**
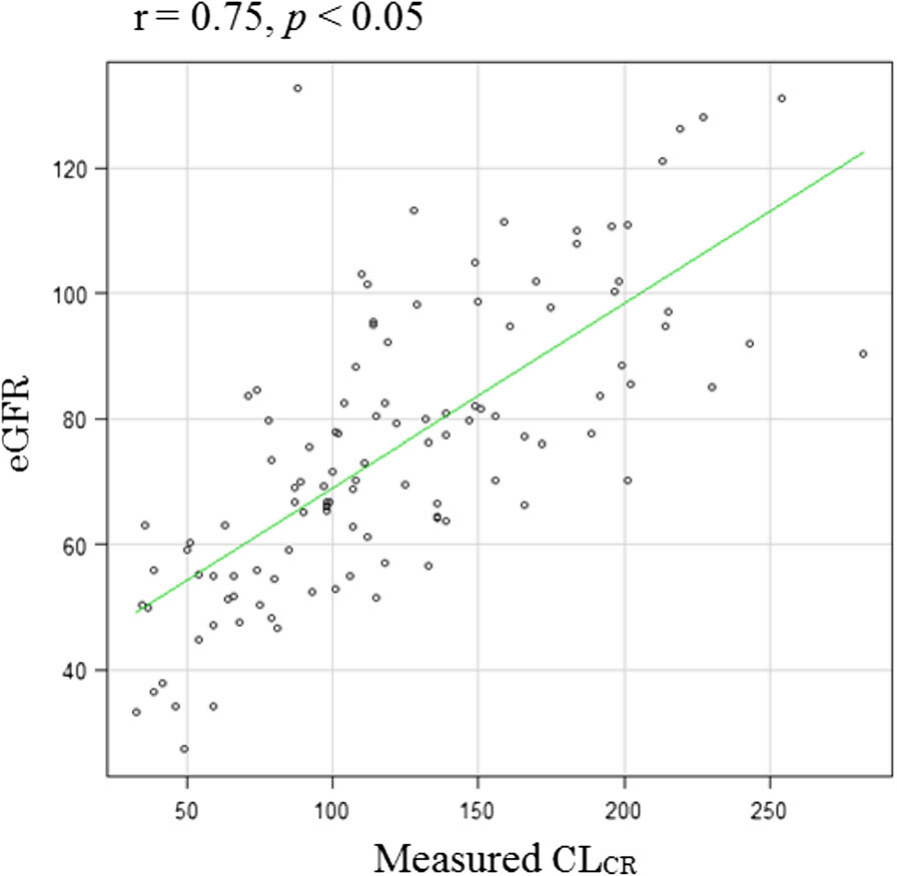
Correlation between the measured creatinine clearance (CL_CR_) and estimated glomerular filtration rate (eGFR). A statistically significant correlation was recognized between the measured CL_CR_ and eGFR with Spearman coefficient of 0.75 (*p* < 0.05)

Analysis to determine the correlation between ARC and eGFR revealed that the eGFR of patients with ARC was significantly higher than that of patients without ARC (*p* < 0.05) (Fig. 1). Moreover, a statistically significant correlation was found between measured CL_CR_ and eGFR, with a Spearman coefficient (*r*) of 0.75 (*p* < 0.05) (Fig. 2). In contrast, the Bland–Altman plots showed that the bias of the two variables was −46.1 mL/min/1.73 m^2^, and the 95 % limits of agreement were −128.9 to 36.7 mL/min/1.73 m^2^.

**Fig. 3:**
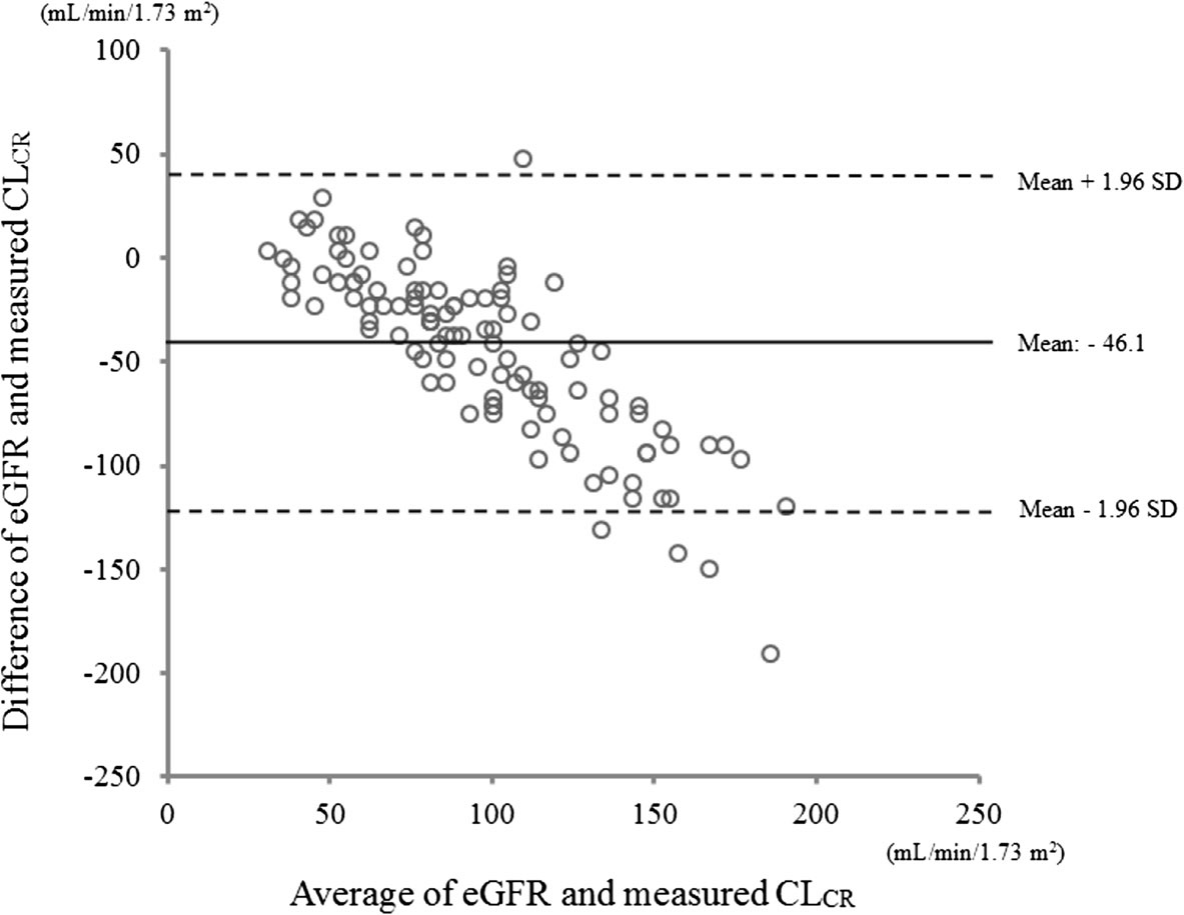
Measures of agreement between the measured creatinine clearance (CL_CR_) and estimated glomerular filtration rate (eGFR). The *solid line* indicates the mean of the difference between the results of the eGFR and measured CL_CR_. The *dashed line* shows the 95 % limits of agreement. Most parts of eGFR tended to underestimate the CL_CR_. In addition, the difference between the eGFR and measured CL_CR_ further increased when the kidney function of the patients improved

Most parts of the eGFR tended to underestimate CL_CR_. In addition, the difference between eGFR and measured CL_CR_ further increased when the kidney function of the patients improved (Fig. 3).

### Prognostic value for ARC

We performed the ROC analysis to evaluate the prognostic value of age and eGFR for ARC. The AUROC of age and eGFR was 0.81 (95 % CI, 0.72–0.89) and 0.81 (95 % CI, 0.73–0.89), respectively. The optimal cutoff value of each factor for ARC was age ≤63 years (sensitivity, 72.1 %; specificity, 82.4 %) and eGFR ≥ 76 mL/min/1.73 m^2^ (sensitivity, 81.4 %; specificity, 72.1 %) (Table 3).

**Table 3:** Augmented renal clearance prediction of age and estimated glomerular filtration rate using the receiver operating curves

	AUROC	95 % CI	Optimal cutoff values	Sensitivity (%)	Specificity (%)	PPV (%)	NPV (%)
Age (years)	0.81	0.72–0.89	63	72.1	82.4	80.4	74.7
eGFR (mL/min/1.73 m^2^)	0.81	0.73–0.89	76	81.4	72.1	74.5	79.5

*eGFR* estimated glomerular filtration rate, *AUROC* area under the receiver operating curve, *CI* confidence interval, *PPV* positive predictive value, *NPV* negative predictive value

## Discussion

To the best of our knowledge, this is the first study that investigated ARC in Japanese adult ICU patients. Our results demonstrate that approximately 40 % of patients who were admitted to our ICU with normal *S*_Cr_ levels on the first hospital day manifested ARC, which was similar to a previous report [3]. Age was identified as an independent risk factor for ARC in multivariate logistic regression analysis in this study, and several previous studies [3, 12] have also shown that ARC is more common in younger patients.

In contrast, no relationship was found between urine output and ARC phenomenon, and the same trend on fluid balance was also shown in this report. We demonstrated that the ARC phenomenon was not simply related to ongoing fluid loading, and a previous study supported this statement [3]. Moreover, with regard to illness severity score, patients with ARC had significantly lower APACHE II scores on admission compared with patients without ARC in the univariate analysis. In contrast, we did not observe the same trend in SOFA scores. The result of the multivariate analysis showed that the different trends in the two severity scores could have been due to the influence of age. The APACHE II score evaluates the illness severity of patients based on physiologic measurements, age, and previous health status [28], whereas the SOFA score was assessed by grading organ dysfunction, not by age [29]. ARC was seen in the younger population; therefore, patients with ARC tend to obtain lower APACHE II scores compared with those of patients without ARC.

Previous studies [12, 30] showed that multi-trauma was a significant risk factor for ARC, but these findings were different from our results. The difference in these findings is likely related to the small sample of patients with severe trauma (only 19 patients) in this single-center study.

The eGFR in this report, which was calculated by using the Japanese equation, was significantly different between patients with and without ARC, and the correlations better represented the true relationship between the measured CL_CR_ and eGFR, with a Spearman coefficient (*r*) of more than 0.7. Although a better correlation was recognized between these variables in this study, eGFR could not detect patients with ARC accurately; eGFR was not considered for ICU patients with severe conditions that influenced renal function because eGFR was principally designed for use in an ambulatory or ward-based setting initially [31, 32]. Therefore, previous reports [12, 13] showed that the derived values from several formulae (Cockcroft–Gault and MDRD) significantly underestimated the CL_CR_, and no eGFR formula accurately identifies ARC in critically ill patients. However, given the better correlation between the measured CL_CR_ and eGFR calculated using the Japanese equation, this study showed that eGFR might be a useful tool for screening Japanese patients with ARC. Because eGFR tended to underestimate the CL_CR_ as shown in the Bland–Altman plots, the eGFR cutoff values for screening ARC were ≥76 mL/min/1.73 m^2^, which was lower than 130 mL/min/1.73 m^2^. In addition, age ≤63 years could also be evaluated for screening simultaneously. After screening patients with ARC, the CL_CR_ should be measured through urine collection formally for modifying the drug dosage as necessary.

This study has some limitations. First, this was a single-center study including a limited number of study participants. Second, this study was not designed to assess ARC after the second hospital day. Although ARC on the first hospital day was strongly associated with higher clearances over a few days, ARC occurring after the second hospital day has been reported [3]. Third, the gold standard for the assessment of renal function is measurement of the urinary or plasma clearance of an ideal filtration marker (such as inulin) [33], but this measurement was not performed in this study. Fourth, because we did not evaluate eGFR, which was calculated by using various formulas (such as Cockcroft–Gault, MDRD, Robert, and CKD-EPI) used worldwide, for identifying ARC in the present study, the best equation for eGFR to identify Japanese patients with ARC is unclear. Fifth, although the creatinine levels were determined by an enzymatic method in the present study, the creatinine levels were determined by other methods such as the Jaffe method in a previous study, which was cited for the present ARC definition. The creatinine levels in serum and urine by the Jaffe method are higher than those by the enzyme method, and CL_CR_ values are affected by these measurement methods [34]. Thus, ARC definition might need to be changed based on the measurement method for creatinine levels. Finally, because this report is a pilot study for ARC in the Japanese population, a validation of the predictive factors for ARC (such as age and eGFR) was not performed. Therefore, further studies are needed to address the limitations of this study.

## Conclusions

This study showed that ARC appeared to be common in Japanese ICU patients with normal *S*_Cr_ levels on the first hospital day, and only age was an independent risk factor for ARC. In addition, not only age but also eGFR calculated using the Japanese equation might be useful as a screening tool for identifying Japanese patients with ARC. Further multicentre studies are needed to obtain precise data regarding ARC in the Japanese population.

## Abbreviations

AKI: Acute kidney injury
APACHE: Acute Physiology and Chronic Health Evaluation
ARC: Augmented renal clearance
AUROC: Area under the receiver operating curve
BSA: Body surface area
CI: Confidence interval
CL_CR_: Creatinine clearance
eGFR: Estimated glomerular filtration rate
GFR: Glomerular filtration rate
ICU: Intensive care unit
IDC: Indwelling urinary catheter
IQR: Interquartile range
ISS: Injury severity score
MDRD: Modification of Diet in Renal Disease
OR: Odds ratio
ROC: Receiver operating curve
*S*_Cr_: Serum creatinine
SD: Standard deviation
SIRS: Systemic inflammatory response syndrome
SOFA: Sequential Organ Failure Assessment
*U*_Cr_: Urinary creatinine
